# A flexible and standalone forward simulation model for laboratory X-ray diffraction contrast tomography

**DOI:** 10.1107/S2053273320010852

**Published:** 2020-09-18

**Authors:** H. Fang, D. Juul Jensen, Y. Zhang

**Affiliations:** a Department of Mechanical Engineering, Technical University of Denmark, Kgs. Lyngby, 2800, Denmark

**Keywords:** 3D grain mapping, diffraction contrast tomography, X-ray diffraction, forward simulation, grain reconstruction

## Abstract

Laboratory X-ray diffraction contrast tomography (LabDCT) has recently been developed as a powerful technique for non-destructive mapping of grain microstructures in bulk materials. As the grain reconstruction relies on segmentation of diffraction spots, it is essential to understand the physics of the diffraction process and resolve all the spot features in detail. To this aim, a flexible and standalone forward simulation model has been developed to compute the diffraction projections from polycrystalline samples with any crystal structure. The accuracy of the forward simulation model is demonstrated by good agreements in grain orientations, boundary positions and shapes between a virtual input structure and that reconstructed based on the forward simulated diffraction projections of the input structure. Further experimental verification is made by comparisons of diffraction spots between simulations and experiments for a partially recrystallized Al sample, where a satisfactory agreement is found for the spot positions, sizes and intensities. Finally, applications of this model to analyze specific spot features are presented.

## Abstract 2

A flexible and standalone forward simulation model has been developed to compute the diffraction projections for laboratory diffraction contrast tomography (LabDCT). The outputs are expected to be of great value for all present users of LabDCT as well as interested new users.

## 1. Introduction   

Non-destructive characterization of grain structures in 3D, resolving the grain sizes, shapes and orientations, provides a versatile tool for improving the understanding of fundamental materials science processes, such as phase transformation, recrystallization and grain growth in polycrystalline materials. Over the past two decades, huge effort has been devoted to the development of a number of such techniques using high-flux X-rays from synchrotron sources (Poulsen & Juul Jensen, 1995 ▸; Yang *et al.*, 2004 ▸; Poulsen, 2012 ▸; Reischig *et al.*, 2013 ▸), including differential aperture X-ray microscopy (DAXM) (Larson *et al.*, 2002 ▸), 3D X-ray diffraction (3DXRD) (Poulsen *et al.*, 2001 ▸; Margulies *et al.*, 2001 ▸; Poulsen & Fu, 2003 ▸) and diffraction contrast tomography (DCT) (Ludwig *et al.*, 2008 ▸; Johnson *et al.*, 2008 ▸). DAXM has been demonstrated for resolving grain orientations and shapes with a resolution <500 nm. 3DXRD and its variants like high-energy X-ray diffraction microscopy and DCT are fast tools for grain mapping with a spatial resolution down to about 1 µm (Offerman *et al.*, 2002 ▸; Schmidt *et al.*, 2004 ▸; King *et al.*, 2008 ▸; Oddershede *et al.*, 2010 ▸; Li *et al.*, 2012 ▸). More recently, dark-field X-ray microscopy has been developed to enable mapping of grains with a spatial resolution of 100 nm by inserting an X-ray objective lens in the diffracted beam to magnify diffraction patterns (Simons *et al.*, 2015 ▸; Jakobsen *et al.*, 2019 ▸; Kutsal *et al.*, 2019 ▸). Besides these techniques, other approaches using synchrotron X-rays, neutrons and electrons for grain mapping at various length scales have been reported (Bernier *et al.*, 2011 ▸; Clark *et al.*, 2012 ▸; Hayashi *et al.*, 2015 ▸; Peetermans *et al.*, 2014 ▸; Raventós *et al.*, 2019 ▸; Liu *et al.*, 2011 ▸).

Although the above-mentioned X-ray techniques are invaluable tools for grain mapping, they require a very brilliant photon beam that is only available at synchrotron sources, which seriously limits the accessibility. To make non-destructive 3D mapping possible at in-house laboratories with daily access, laboratory X-ray diffraction contrast tomography (LabDCT) has been developed based on ideas from synchrotron DCT (King *et al.*, 2013 ▸, 2014 ▸; McDonald *et al.*, 2015 ▸; Holzner *et al.*, 2016 ▸). LabDCT adopts a conical polychromatic X-ray beam generated from a conventional X-ray tube to illuminate a sample with a typical size of hundreds of microns to millimetres. The grain structure of the sample is reconstructed from a series of LabDCT diffraction images recorded as the sample rotates over 360°. The LabDCT technique has proven to be a powerful tool for non-destructive 3D grain mapping for polycrystalline powders, minerals and metals (McDonald *et al.*, 2017 ▸; Pankhurst *et al.*, 2019 ▸; Sun *et al.*, 2019 ▸, 2020 ▸).

Today, 3D reconstructions of grain orientations, positions and shapes are routinely available using the reconstruction software *GrainMapper3D* developed by Xnovo Technology ApS (Bachmann *et al.*, 2019 ▸; Oddershede *et al.*, 2019 ▸). A fast geometric indexing approach is used to reconstruct the grains based on pre-processed and segmented diffraction spots. A forward projection model has been implemented in *GrainMapper3D* (version 2.0 or higher) to compute the shape of the diffraction spots based on the reconstructed grain structure. By comparing the simulated and experimentally observed diffraction patterns, any shifts and tilts of the detector can be minimized and the reconstruction can then be further optimized (Niverty *et al.*, 2019 ▸). Besides *GrainMapper3D*, an iterative tomographic reconstruction approach is also reported for grain reconstruction based on a projection model (van Aarle *et al.*, 2015 ▸). Although these forward simulation models are able to compute spot positions and shapes, they mainly serve as reconstruction tools and are lacking in aspects such as detailed descriptions of the principles and implementation of the model, capabilities to compute spot intensities and quantitatively compare spot features, including sizes, shapes and intensities, between simulation and experimental data. All these aspects are important for understanding the physics of the diffraction process and optimizing LabDCT experiments.

In the current work, we present a flexible and standalone forward simulation model to compute LabDCT diffraction projections. This model provides physical insights into the diffraction process and all the details about each diffraction spot, including diffracting X-ray energies, *hkl* index, position, size, shape and intensity; based on this, detailed diffraction information from individual grains can be readily obtained. This model can thus be used as a virtual tool to predict spot features for samples with different grain structures under different LabDCT experimental conditions, and thereby used to optimize any given experiment. Compared with other forward simulation models (*e.g.* the one in *GrainMapper3D*), our model has the advantage of being transparent, with detailed descriptions of both model principles and implementation, and serving as a tool to analyze all the details of diffraction spots. In Section 2, we present the principles and implementation of the forward simulation model. In Section 3, we verify the accuracy of the model using both a virtually rendered and a real sample. The virtual sample is used first to simulate LabDCT diffraction images, and then as a ground truth to compare with the volume reconstructed using the simulated diffraction images. A 3D grain structure in a real Al sample characterized by a LabDCT experiment is then used to verify the model further by comparing all features of the simulated diffraction spots with the corresponding experimental ones. In Section 4, we present examples of the application of the model, including retrieving experimental spots and analyzing spot details.

## 2. Forward simulations of projections for LabDCT   

LabDCT utilizes a conical polychromatic X-ray beam from a laboratory X-ray tube, which is different from both a parallel monochromatic beam used for synchrotron DCT/3DXRD and a focused polychromatic X-ray beam used for DAXM (Lauridsen *et al.*, 2001 ▸; Suter *et al.*, 2006 ▸; Sørensen *et al.*, 2012 ▸; Sharma *et al.*, 2012 ▸; Schmidt, 2014 ▸; Larson & Levine, 2013 ▸). The principle of LabDCT is therefore different from any of the synchrotron techniques. In this section, we will first present a detailed description of the principle of LabDCT and the forward simulation. Then, we show the implementation of the forward model for simulating LabDCT diffraction projections using a new polyhedron meshing based approach.

### 2.1. Principle of LabDCT and the forward simulations   

**Figure 1:**
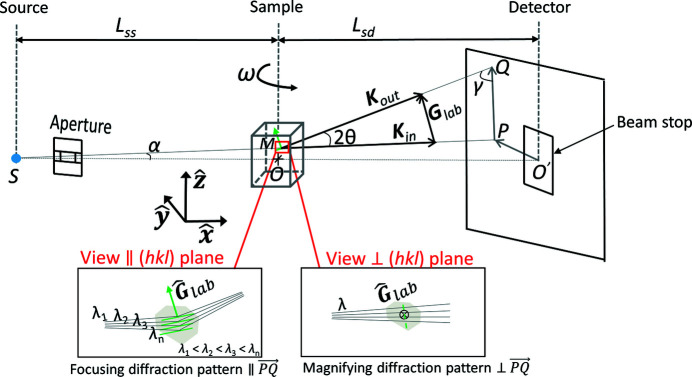
Schematic view of the LabDCT setup in a laboratory coordinate system (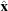, 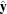, 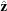). Laue focusing occurs from a volume element of a grain, centered at position *M*, in a polycrystalline sample illuminated by a cone-shaped polychromatic X-ray beam from point *S*. The detector is placed perpendicular to the horizontal line in the downstream transmitted direct beam. The diffraction plane defined by the incoming wavevector **K**_in_ and scattered wavevector **K**_out_ determines the position *Q* on the detector for the diffracted beam. **G**_lab_ is the scattering vector and its projection on the detector is along 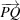. The sample rotation angle is denoted as ω. *L*_ss_ is the sample-to-source distance and *L*_sd_ is the sample-to-detector distance. The zoom-in views (at the bottom) illustrate that the volume element focuses the incoming conical X-rays with a small range of wavelengths between λ_1_ and λ_*n*_ onto the detector along 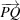, while the diffraction pattern is magnified perpendicular to 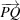.

A schematic of the LabDCT setup is shown in Fig. 1 ▸. The system is defined in a laboratory coordinate system: 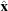 is along the incoming horizontal X-ray beam, 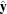 is transverse to the beam in the horizontal plane, 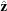 is along the vertical axis that is perpendicular to the beam, and *O* (0, 0, 0) is the origin. The sample is mounted on a rotation stage, having the rotation axis coincident with the 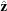 axis, and placed between an X-ray source and a 2D detector. The X-ray source is assumed to be a point source at position *S* (−*L*_ss_, 0, 0) as its size is negligible compared with *L*_ss_ or *L*_sd_ (*L*_ss_, sample-to-source distance; *L*_sd_, sample-to-detector distance). An aperture is placed close to the source to confine the beam. The detector is placed perpendicular to the horizontal beam at a distance of *L*_sd_ from the origin and *O*′ (*L*_sd_, 0, 0) is denoted as the detector center. The direct transmitted beam is blocked by a beam stop, while the diffracted signals are recorded by the outer area of the detector. A complete LabDCT data set is obtained by collecting diffracted projections for each rotation step from a full 360° rotation around the 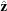 axis with a predetermined step size.

At a given rotation angle, ω, for a volume element *V*_pol_ centered at a position *M* (*x*_m_, *y*_m_, *z*_m_) within the sample with a given crystal structure, its lattice plane (*hkl*) can be considered as a mirror that focuses the incoming X-rays with different wavelengths (λ_1_, λ_2_, λ_3_…λ_*n*_) onto the detector (see Fig. 1 ▸). The diffraction event thus occurs based on a Laue focusing effect, rather than the standard Bragg or Laue diffraction. It has to be noted that the beam is only focused along the direction perpendicular to the (*hkl*) plane (that is along 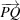 on the detector in Fig. 1 ▸). Within the plane, the incoming beam keeps its divergence while being diffracted and leads to a geometrical magnification in the direction parallel to the (*hkl*) plane (that is perpendicular to 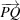 on the detector in Fig. 1 ▸) with a factor of (*L*_sd_ + *L*_ss_)/(*L*_ss_ + *x*_m_). As a result, the diffraction spot on the detector has an elliptical shape and its center *Q* (*L*_sd_, *y*_det_, *z*_det_) can be determined as follows.

The scattering vector **G**_lab_ at the center of mass of *V*_pol_ defined in a laboratory coordinate system for the (*hkl*) plane can be determined as
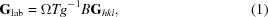
where Ω is a matrix transforming a rotated system to the laboratory system, *T* is a matrix transforming a sample system to the rotated system, 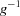 is a matrix transforming a Cartesian crystal system to the sample system, *B* is a matrix transforming a reciprocal space to the Cartesian crystal system, and 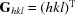. The detailed formulations of these transformation matrices are given in the work of Poulsen (2004 ▸).

The incoming wavevector **K**_in_ of the diffraction event can be expressed as
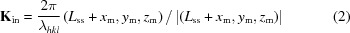
where λ_*hkl*_ is the photon wavelength that fulfills Bragg’s law and reflects at the center of *V*_pol_. The Bragg angle θ is now calculated as
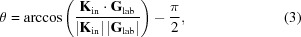
based on which the λ_*hkl*_ can be determined according to Bragg’s equation 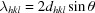, where *d_hkl_* is the lattice spacing of the (*hkl*) planes and *d_hkl_* = 2π/|**G**_lab_|. The scattered wavevector **K**_out_ can be expressed as 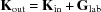.

The projection of the transmitted incoming beam on the detector *P* (*L*_sd_, *y*_p_, *z*_p_) is given by 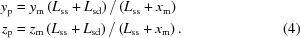
According to the law of sines for the triangle 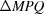, the length of the diffraction displacement *L*_diff_ (*PQ* in Fig. 1 ▸) can be calculated: 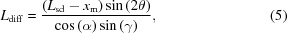
where α = 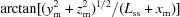 is the angle between 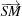 and 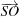 and γ is the angle between 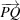 and **K**_out_ (see Fig. 1 ▸). The γ can be calculated as
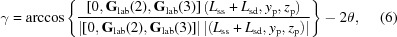
where (*L*_ss_ + *L*_sd_, *y*_p_, *z*_p_) is the vector 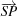 and [0, **G**_lab_(2), **G**_lab_(3)] is a vector parallel to 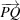. Now the position *Q* (*L*_sd_, *y*_det_, *z*_det_) can be determined with 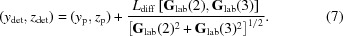



### 2.2. Implementation of the forward simulations   

A polyhedron meshing based model is developed to simulate the diffraction spots from individual grains. By sub­dividing each grain into many small polyhedral volumes and treating each polyhedron independently using the method described above, the 3D grain shape can be accurately depicted by the resolved diffraction spot even for the very complex one. This novel polyhedron meshing based approach has the advantage of conforming to the grain boundaries, thereby avoiding ‘staircase’ artifacts inherent to voxelized grids, which are generally used in other models. The details of the model are described as follows.

**Figure 2:**
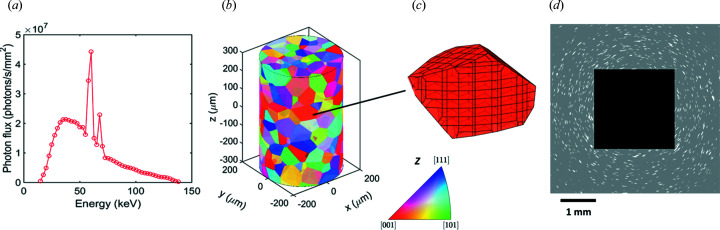
Illustrative workflow of the forward simulations of projections for an Fe sample with pre-defined 3D grain structure. (*a*) Spectrum of the X-ray source using a tungsten target at an acceleration voltage of 140 kV. (*b*) The 3D grain structure of a virtually rendered cylindrical sample (*D* × *H* = 400 × 600 µm) consisting of 666 grains with an average grain size of 58.7 µm. Grain orientations are randomly generated and the grains are colored according to their orientations along the *z* direction [see the color code in the inverse pole figure (IPF)]. (*c*) Polyhedral mesh of a grain. (*d*) Simulated projection at a rotation angle ω = 0° and *L*_ss_ = *L*_sd_ = 11.0 mm (the central black region represents the beam stop).

First X-ray spectra at different electron accelerating voltages from an X-ray source were generated according to the work of Boone & Seibert (1997 ▸). An example X-ray spectrum from a tungsten anode at an acceleration voltage of 140 kV is shown in Fig. 2 ▸(*a*), which should be regarded as an approximation and can be easily corrected once an actual source spectrum is available. The input 3D grain structure can be either virtually rendered or experimentally characterized data sets. Fig. 2 ▸(*b*) shows one example of a virtually rendered 3D volume generated based on Voronoi tessellations using the *mpt3* toolbox (Herceg *et al.*, 2013 ▸). A 3D polyhedral mesh is applied for each grain in the input structure. Each polyhedron belongs to only one grain, *i.e.* no polyhedron crosses a grain boundary. An example of a meshed grain can be seen in Fig. 2 ▸(*c*), where the grain is divided into 269 polyhedral elements with an average size of 12.5 µm.

To simulate a projection at a certain rotation angle ω, diffraction events are calculated grain by grain, polyhedron by polyhedron, and *hkl* by *hkl*. The intensity of the diffraction spot *I*_spot_ for each polyhedron with volume *V*_pol_ can be calculated by the following equation [adapted from Als-Nielsen & McMorrow (2011 ▸), Warren (1990 ▸)]: 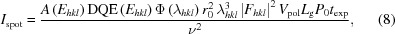
where 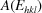 is the attenuation factor due to sample absorption for photons with energy *E_hkl_*, 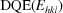 is the detective quantum efficiency (DQE) of the detector system for photons with energy *E_hkl_*, 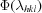 is the incident flux of photons with wavelength λ_*hkl*_, *r*_0_ is the Thomson scattering length and has a value *r*_0_ = 2.82 × 10^−15^ m, *F_hkl_* is the structure factor of the *hkl* reflection, *L*_g_ is the Lorentz factor, *P*_0_ is the polarization factor and is given by 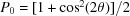, *t*_exp_ is the exposure time for each projection and *v* is the volume of the unit cell. The sample shape has to be known for deriving 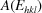. We present a solution of 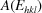 for a cylindrical sample in Appendix *A* [App appa]. If the sample shape is irregular but can be well approximated by a cylinder, this approach also applies. 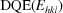 varies with specific detector systems and experimental conditions. In Appendix *B* [App appb] we present details for calculating 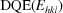 for a scintillation detector using a CsI scintillator with a thickness of 150 µm at zero spatial frequency.

In general the Lorentz factor accounts for the way reflections are integrated. For monochromatic diffraction of single crystals, the Lorentz factor 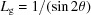 accounts for the time that each reflection is in the diffraction condition (Als-Nielsen & McMorrow, 2011 ▸); for polychromatic Laue diffraction, the Lorentz factor 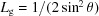 or 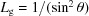 [it does not matter which when relative intensities are considered (Sakamaki *et al.*, 1980 ▸; Lange, 1995 ▸)] accounts for how much of the wavelength range an infinitely small reflection cuts through as a function of θ. However, the present Laue focusing case is different from both these cases. Since the lattice plane acting like a mirror focuses the incoming X-rays at different incident angles and with different energies, it can be considered as a case of parallel X-rays with a single energy, *i.e.* the monochromatic case. On the other hand, different lattice planes diffract X-rays with different energies, which can be considered as polychromatic Laue diffraction. To test which treatment is more suitable, we performed simulations using these two different expressions of Lorentz factors and compared them with the experimental data. Results show that taking the Lorentz factor as 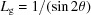 gives a much better correlation between simulation and experimental data. Thus, we use the expression for the monochromatic case in the present study.

To account for the point spread nature of the interaction between photons and the detector, the intensity *I*_spot_ is distributed to an array of pixels (*p*_min,1_ × *p*_min,2_) with the center position determined according to equation (7). Here, the distribution weight matrix is generated by convolution of a linear motion filter (with the moving direction parallel to the projection of the *hkl* reflection on the detector) and a Gaussian filter, which leads to an anisotropic point spread with larger weights assigned along the direction perpendicular to 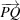 (accounting for the magnification effect) while smaller weights are assigned parallel to 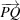 (accounting for the Laue focusing effect). The sizes of both the motion filter and the Gaussian filter are determined by the polyhedron size *d*_pol_ and the pixel size of the detector *d*_pixel_, as 2*d*_pol_/*d*_pixel_. The dimensions of the resulting convolution matrix now determine the values of *p*_min,1_ and *p*_min,2_.

To simulate the projection, a 2D detector with 2032 × 2032 pixels centered at (1016, 1016) with an effective pixel size of 3.36 µm is used, which is about the same as that used in the commercial LabDCT system. For each pixel on the detector, the intensity 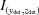 is summed for all diffraction signals arriving at this pixel. A constant background intensity is added to each pixel to mimic the inelastic scatterings from the sample. Fig. 2 ▸(*d*) shows a diffraction projection for the 3D grain structure shown in Fig. 2 ▸(*b*).

Generally, a smaller polyhedron size with a more isotropic shape will result in a better resolved spot shape. Since the polyhedra are represented by Voronois that are generated by placing seeding points inside every grain, the number of polyhedra in each grain is decided by the number of seeding points. This means that the average polyhedron size decreases with increasing number of seeding points. It should be noted that sizes of polyhedra in the same grain may vary; in particular, those touching grain boundaries vary more as they must adapt to conform to the grain boundaries. In the present study the seeding points are homogeneously generated with their number in 1D determined according to the grain diameter divided by the pixel size of the detector. To balance the accuracy and the computing efficiency, polyhedra with an average polyhedron size smaller than 12.5 µm resulting from this meshing algorithm are recommended. In addition to the size, the polyhedron shape can influence the local intensity distribution, but it has less impact on the overall shape and intensity of the spots since polyhedra do not significantly deviate from isotropic shapes when a fine mesh is used. A complete LabDCT data set is then generated by computing all the projections for all the rotation angles. Based on the simulation, the properties of individual diffraction spots are also determined, including the locations, sizes, shapes, integrated intensities and X-ray energies, as well as information about which *hkl* reflection is from which grain. The current forward simulations are coded in *MATLAB* and the projections are exported in the form of 16-bit gray images. The codes can be found at https://github.com/haixingfang/LabDCT-forward-simu-model.

## 3. Model validation   

### 3.1. Validation using a virtual grain structure   

As a first test of the proposed forward simulation model, a virtually rendered 3D grain structure is used as input for the simulation. From the simulated LabDCT diffraction projections, standard routines are employed to reconstruct the grain structure [here we use those implemented in *GrainMapper3D* (Bachmann *et al.*, 2019 ▸)]. A good agreement between the input and the reconstructed grain structures would validate the forward simulation.

**Figure 3:**
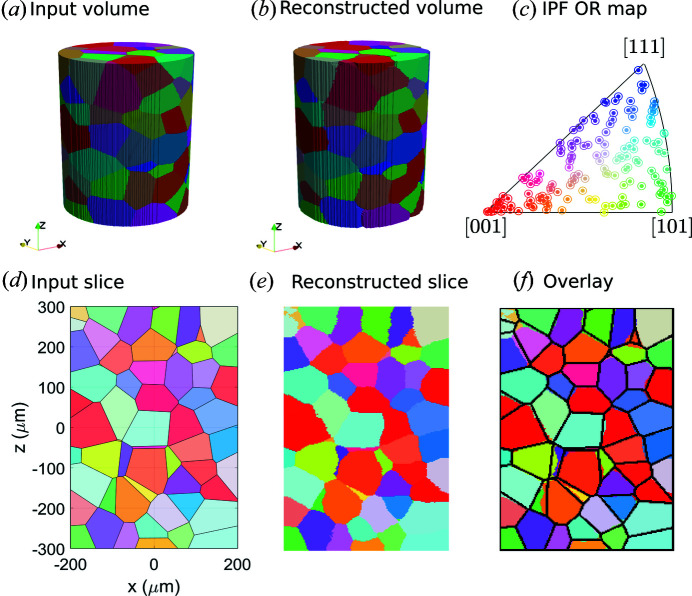
Validation of the model using a virtually rendered 3D grain structure of iron. (*a*) Input 3D grain structure for simulating diffraction projections. (*b*) Reconstructed grain structure from the simulated projections. (*c*) Crystallographic orientations (OR) along *z* of the input grains (marked by dots) and the reconstructed grains (marked by circles). (*d*) and (*e*) show 2D *xz* slices taken from the middle of the input and reconstructed grain structures, respectively. (*f*) An overlay of the reconstructed 2D slice with the black lines showing the grain boundary positions of the input grain structure.

Fig. 3 ▸(*a*) shows the input grain structure of iron. The input consists of 144 grains with an average size of 98.7 µm. The standard deviation of the grain size distribution is 11.0 µm. Using the forward simulation procedure described above (here grains were meshed into polyhedra with sizes of 9.9 ± 0.7 µm), 181 diffraction images with a rotation interval of 2° were computed, which are used subsequently to reconstruct (restore) the 3D grain structure using the commercial software *GrainMapper3D*. Good agreement between the reconstructed and the input structures is obtained [see Figs. 3 ▸(*a*)–3 ▸(*c*)]. This is more clearly visible in 2D cross sections, as shown in Figs. 3 ▸(*d*)–3 ▸(*f*). The total number of 3D reconstructed grains is 144 and the average grain size is found to be 98.5 µm with a standard deviation of 13.0 µm for the grain size distribution, which are all in excellent agreement with the input. An even more critical validation can be obtained by comparing directly the orientations of individual grains and grain boundary positions. This detailed comparison shows an agreement better than 0.03° in orientation determination. 94% of the voxels are fully matched and 99% of the voxels deviate by no more than 2 voxels, whereas a deviation of up to 8 voxels is observed for some grain boundary segment positions. It has to be noted that the quality of the reconstructed grain structure not only depends on the forward simulation but also on the parameters used for both spot segmentation and grain reconstruction with *GrainMapper3D*. The latter is suggested to play a more dominant role than the former. Overall, it can be concluded that the proposed simulation model performs adequately.

### 3.2. Validation using an experimentally characterized partially recrystallized structure   

Another way to validate the model is to use the grain structure from a LabDCT/*GrainMapper3D* measured/reconstructed sample as input for the forward simulation and then compare the simulated and measured diffraction spots directly. A good agreement between the two would further validate that the input X-ray spectrum is close to that generated in reality by an X-ray tube and that independent treatment of the polyhedron mesh for each grain is a good approach for simulating diffraction spots.

#### 3.2.1. Experimental LabDCT measurements   

LabDCT measurements were performed using a partially recrystallized pure aluminium (99.996 wt% Al) sample. The sample (6.0 × 4.0 × 1.3 mm) was cut from a 12% cold-rolled Al plate, ground and electro-polished to remove the cutting damage. A Vickers hardness indent was made on the surface plane defined by the rolling direction (RD) and the transverse direction (TD) to stimulate nucleation of new grains upon annealing. The sample was annealed to partial recrystallization. Details on heat treatment *etc*. can be found in the work of Xu *et al.* (2017 ▸), Zhang *et al.* (2020 ▸).

The LabDCT measurements were performed using a Zeiss Xradia 520 Versa X-ray microscope. The parameters of the detector are the same as described above. The scanning was performed with the Laue focusing geometry, *L*_ss_ = *L*_sd_ = 14.0 mm. The accelerating voltage was 150 kV and the exposure time for each projection was 600 s. A total of 181 diffraction projections were acquired by rotating the sample 360° with an interval of 2°. Additionally, 1601 absorption contrast tomographic projections were sequentially collected with an exposure time of 1.2 s to reconstruct the sample gauge volume. The grain reconstruction was performed with *GrainMapper3D* version 2.1 by indexing the first three {*hkl*} families and subsequently including fitting of the detector position to optimize the final reconstruction. The grain structure was reconstructed with a voxel size of 2.5 µm.

#### 3.2.2. Experimental results and comparison with the simulations   

**Figure 4:**
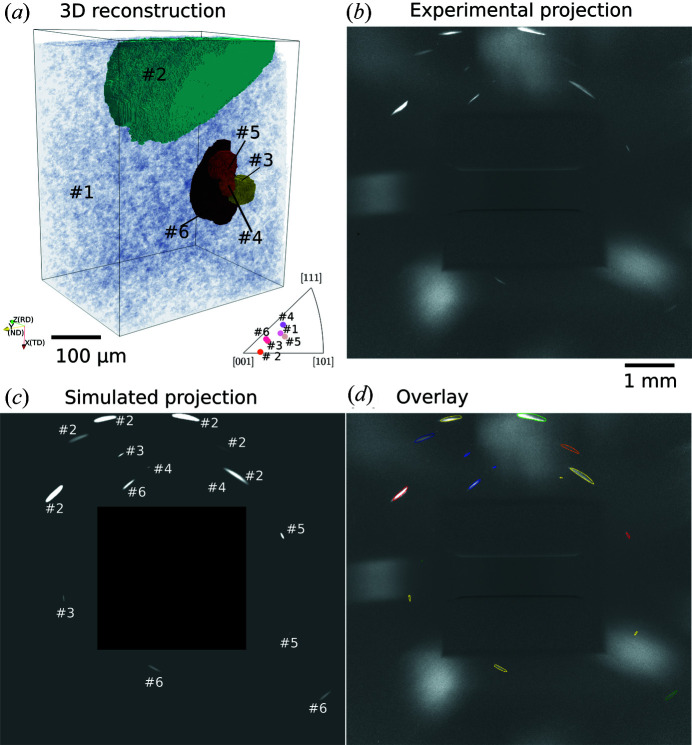
(*a*) 3D visualization of reconstructed grains (shown in random colors) in a partially recrystallized Al sample determined by LabDCT measurements, with an inverse pole figure (IPF) triangle (along the sample ND) showing the crystallographic orientations of the grains. Grain #1 is deformed and made semi-transparent for visualization. (*b*) An experimental diffraction projection at the rotation angle of −146°. (*c*) The corresponding simulated projection with grain numbers marked for each diffraction spot. (*d*) An overlay of the outlines of the simulated spots in (*c*) onto (*b*). The outlines in (*d*) are colored according to the {*hkl*} families: red {111}, green {002}, blue {022}, yellow {113}, olive {133}, purple {024}, navy {224}, orange {115}.

There are six reconstructed grains with sizes >30 µm in the sample. Details of the six grains are listed in Table 1 ▸. A 3D visualization of the reconstructed grains is shown in Fig. 4 ▸(*a*). Grain #1 is in a deformed/recovered state with a significant spread of orientations and made semi-transparent in Fig. 4 ▸(*a*) for visualization. All the other five grains are recrystallized and reconstructed with a relatively high average completeness across all the voxels within the same grain (>75%). In *GrainMapper3D* the completeness of each voxel is defined by the number of indexed reflections as the fraction of the theoretical number of reflections computed for this voxel (Bachmann *et al.*, 2019 ▸).

We used the reconstructed grain structure shown in Fig. 4 ▸(*a*) as input for the simulation. The five recrystallized grains were meshed into polyhedra with average sizes ranging from 5.5 to 10.9 µm. Due to the lack of an X-ray spectrum for the acceleration voltage of 150 kV, we used the profile of the X-ray spectrum at the acceleration voltage of 140 kV for the simulation, which is expected to generate negligible difference for the outcome. Fig. 4 ▸(*b*) shows an example of the experimental projection at a rotation angle ω = −146° and the corresponding simulated projection is shown in Fig. 4 ▸(*c*). The large ‘blobs’ seen in Fig. 4 ▸(*b*) are reflections from the deformed grain and are not considered in the simulations. An overlay of the outer edges of the simulated diffraction spots on the experimentally determined ones is shown in Fig. 4 ▸(*d*). The figure shows that all the diffraction spots are well reproduced in terms of positions, shapes and sizes, which further validates our forward simulation model.

**Figure 5:**
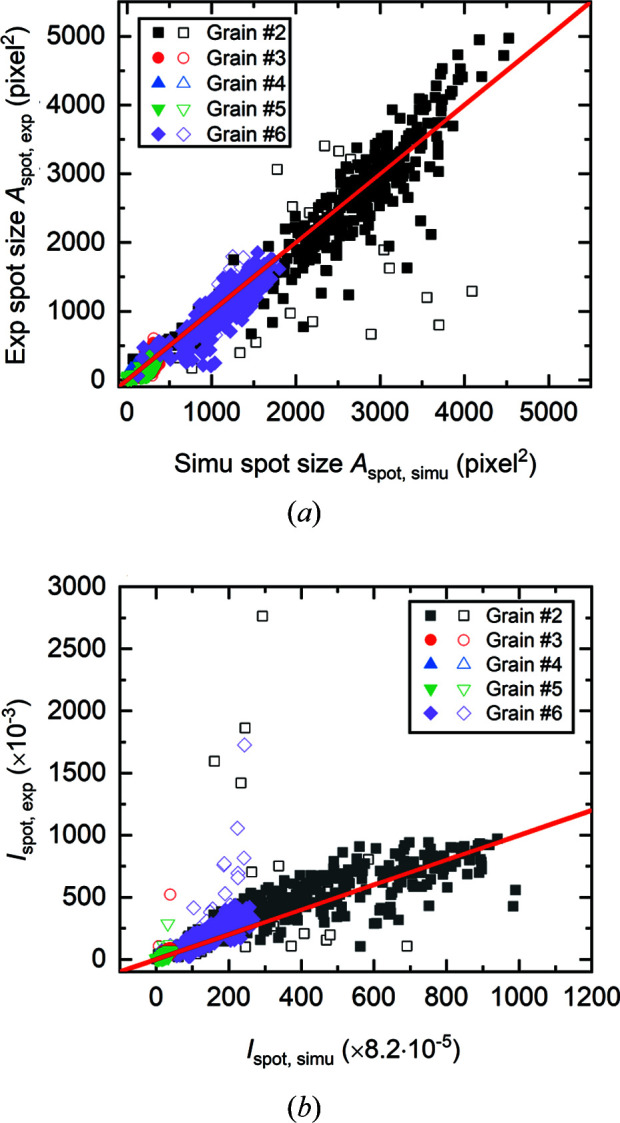
Comparisons of (*a*) spot sizes *A*_spot_ and (*b*) spot integrated intensities *I*_spot_ between simulated (Simu) and experimentally (Exp) observed diffraction spots for the first four {*hkl*} families. The lines in (*a*) and (*b*) indicate that the simulated spot sizes are equal to the experimental ones and that the scaled integrated intensities of the simulated spots are equal to those of the experimental ones, respectively. The closed symbols represent ‘good’ spots and the open symbols stand for ‘bad’ spots.

Further validation is made by comparing the size and intensity of the simulated and experimental diffraction spots in the whole series of projections for a full rotation of 360°. Here only the spots from the first four strongest {*hkl*} families, which are typically the important ones for grain reconstruction, are considered. The total number of diffraction spots that have intensities distinguishable from the background in the experimental projections is listed in Table 1 ▸. More spots are observed for larger grains (see Table 1 ▸), agreeing with their higher completeness values. As the absolute intensities of both spots and backgrounds are very different between simulation and experimental data, spot segmentations were performed in different ways for the two types of data: the average value of the thresholds determined by Otsu’s method (Otsu, 1979 ▸) and the unimodal background-symmetry method (DIP*image* 2.9 toolbox; DIP*image*, 2017 ▸) were applied to segment each simulated spot and determine its size. For each experimental spot a single threshold value (which varies from spot to spot) was used and for the segmentation the corresponding dilated simulated spot was employed as a mask. Notably each spot is segmented independently. Based on the features of the segmented experimental spots, we identified two types of spots: one is well segmented and not overlapped with other spots, referred to as ‘good’ spots here; the other is overlapped with other spots or has problems with segmentation due to a too low contrast compared with the background, referred to as ‘bad’ spots here. Fig. 5 ▸ shows a detailed comparison between the simulated and experimentally observed diffraction spots, including all the ‘good’ and ‘bad’ ones. It can be seen from Fig. 5 ▸ and the grain size data given in Table 1 ▸ that a strong correlation exists between the grain size and the spot size as well as the intensity. Both Figs. 5 ▸(*a*) and 5 ▸(*b*) show that the majority of the spots follow well the red lines with a slope of 1, indicating that the simulations are in good agreement with the experimental data. In Fig. 5 ▸(*b*) the integrated intensities of spots from the simulations are scaled by dividing with a constant of 12.20. It should be noted that this constant does not have any physical meaning as the observed experimental integrated intensities are simply gray values of spot pixels on the projections rather than actual photon counts. Fig. 5 ▸(*b*) thus documents that the relative spot intensities can be well predicted by the current model. Both Figs. 5 ▸(*a*) and 5 ▸(*b*) show that the outliers far from the red lines are mainly the ‘bad’ spots. In particular, in Fig. 5 ▸(*b*) most of the ‘bad’ spots are located above the red line, indicating that they are overlapped with other spots. Besides the outliers, the data points for the ‘good’ spots are also scattered around the red lines, which can be due to the non-uniform experimental beam profile and its variation over time, noise of the experimental measurement, as well as, to a certain extent, the imperfect grain reconstruction. Altogether, the results demonstrate that the polyhedron meshing based approach predicts satisfactorily the sizes and intensities of individual spots.

## 4. Application of the forward simulation model   

In a recent study (Hovad *et al.*, 2020 ▸), we have demonstrated that the simulated LabDCT diffraction projections can be used as input to train a deep learning algorithm to identify the diffraction spots in experimental images. Here we will show two other application examples of the model: (i) retrieving all the experimental diffraction spots; (ii) analyzing spot intensities as a function of photon energy.

### 4.1. Retrieving experimental spots   

**Figure 6:**
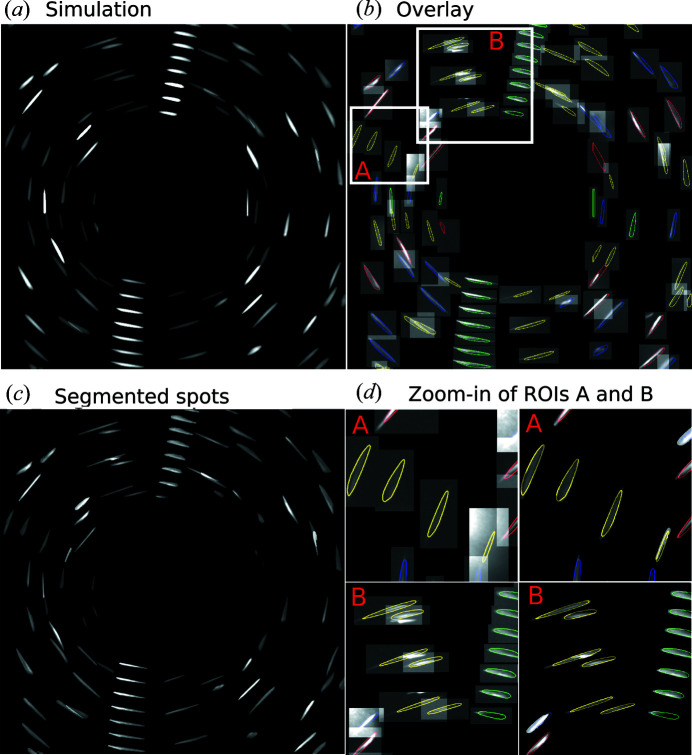
Images summarizing all diffraction spots from grain #2 [see Fig. 4 ▸(*a*)] in all projections from −180 to 0° with an interval of 4°. (*a*) Simulation; (*b*) overlay of outer edges of the simulated spots onto the corresponding cropped regions of the experimental projections; (*c*) segmented diffraction spots using the simulated spots as masks; (*d*) zoom-in views of regions of interest [ROIs A and B in (*b*)] show that (A) weak and (B) partially overlapped diffraction spots can be well retrieved. The outlines are colored according to the reflections of the {*hkl*} families: red {111}, green {002}, blue {022}, yellow {113}.

We can combine the forward simulation with LabDCT experiments to retrieve all the experimental diffraction spots from individual grains and then analyze the spot information. For example, using the 3D grain structure in Fig. 4 ▸(*a*) as input, all the simulated diffraction spots from grain #2 can be readily obtained and summarized in one image [see Fig. 6 ▸(*a*)]. The simulated spots can then be used to identify the locations of all the corresponding experimental spots [see Fig. 6 ▸(*b*)] and used as masks to segment the experimental spots [Fig. 6 ▸(*c*)]. Thus, we can overcome challenges in segmenting weak as well as overlapped spots. Fig. 6 ▸(*d*) shows examples of segmenting these two types of spots, those that are weak (in region A) and those touching other spots (in region B). The retrieval of all the experimental spots is important for analysis of spot details, and a further analysis could uniquely identify the ‘good’ and ‘bad’ spots and separately quantify spot features like size, intensity *etc*. (see Fig. 5 ▸ for example) based on comparison of spot features between the simulation and experiment. However, this is outside the scope of the present work.

**Figure 7:**
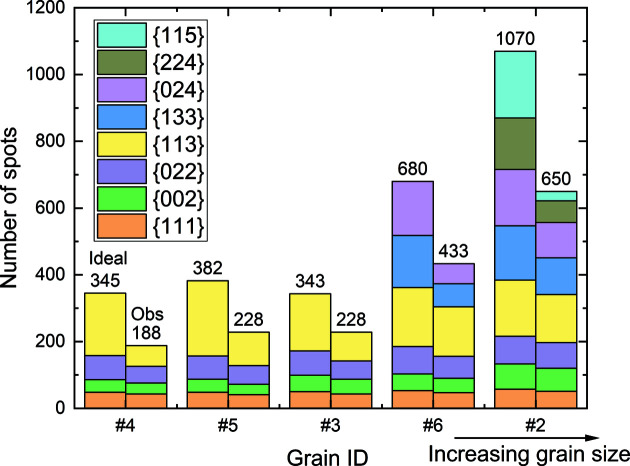
Number of diffraction spots for each {*hkl*} family of each grain. The two columns are: left – number of theoretically predicted spots that hit onto the effective area of the detector; right – number of experimentally observed spots.

We can also readily compute the theoretical number of spots for each grain using our forward simulation model. Taking the recrystallized grains in the Al sample as an example, we plot the theoretical number of spots and the number of experimentally observable spots in Fig. 7 ▸. The figure clearly shows the correspondence for the number of spots from each {*hkl*} family between the simulation and experimental observation as a function of grain size – both numbers decrease with decreasing grain size. Taking the ratios between the number of experimentally observed and theoretical spots, we can determine the values of theoretical maximum completeness. Obviously, the values decrease with decreasing grain size and the decrease is even greater when more {*hkl*} families are considered. For example, the values of theoretical maximum completeness are 0.80 for grain #4 (40.6 µm) and 0.94 for grain #2 (248.5 µm) when the first three {*hkl*} families are considered. When the first four {*hkl*} families are considered, the values decrease to 0.54 for grain #4 and 0.91 for grain #2.

### 4.2. Spot intensity as a function of {*hkl*} family and X-ray energy   

Both the lattice plane and the photon energies for each diffraction spot can be determined from the forward simulation. By correlating this information to the experimental spot intensity, the relationship between the photon energy/{*hkl*} family and the spot intensity, as well as its dependence on grain size can be studied. This is essential for understanding how diffraction events for a specific grain are affected by the polychromatic laboratory X-ray source.

**Figure 8:**
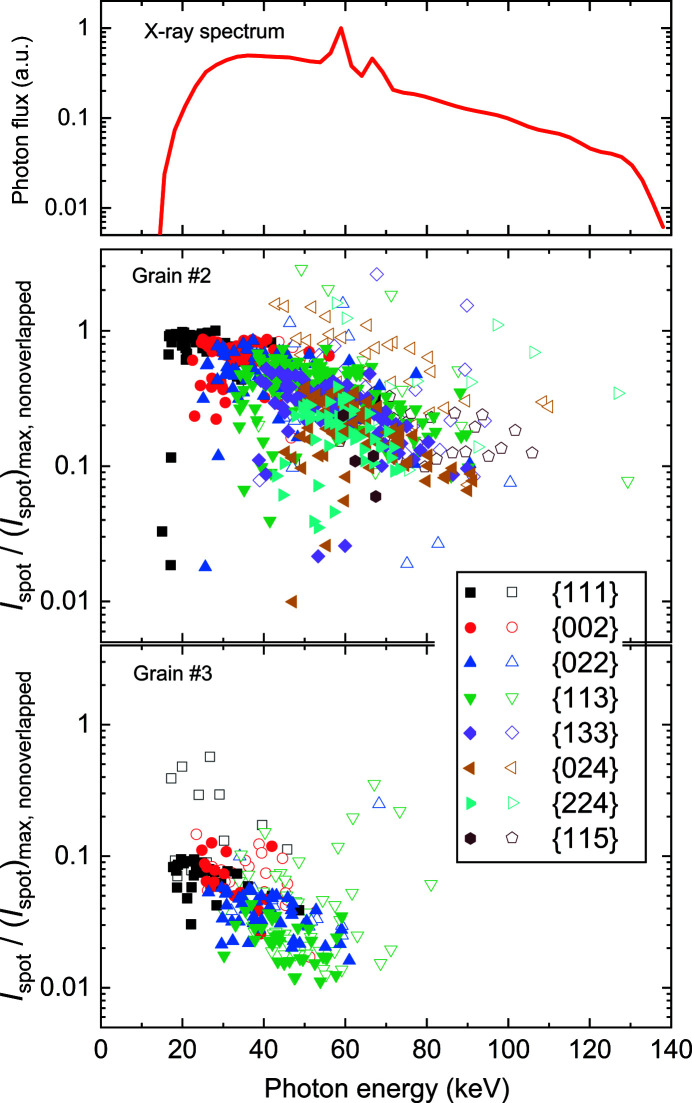
Plots of the spot integrated intensities determined from the experimental data as a function of the photon energy obtained from the simulations for all the experimental spots of grains #2 and #3 in the Al sample. The closed symbols represent ‘good’ spots and the open symbols are for ‘bad’ spots. The intensities are scaled by dividing by the maximum integrated intensity of all the experimentally observed ‘good’ spots. The profile of the X-ray spectrum used in the simulations is also plotted and normalized with respect to its maximum. The average standard deviations of the spot energies are 2.4 keV for grain #2 and 0.6 keV for grain #3.

Fig. 8 ▸ shows the relationship between the normalized integrated spot intensities and the photon energies as well as {*hkl*} families for a large grain (grain #2, 248.5 µm) and a smaller grain (grain #3, 71.2 µm) from the Al sample shown in Fig. 4 ▸. Here only the photon energy averaged over the whole grain is used for each diffraction spot. The figure shows that for the majority of the spots the intensities for both grains match reasonably well with the expected spectrum profile of the X-ray source. Since the ‘bad’ spots are mainly overlapped with others, they have abnormal high intensities and are thus located apart from the majority. Overall, spots diffracting from higher-order {*hkl*} planes are from higher photon energies. For the large grain, spots up to the tenth {*hkl*} family are detectable and the photon energies are mainly distributed in the range 15–80 keV (96% of the total number of 525 ‘good’ spots). For the small grain, only the first four *hkl*-index spots can be identified and the corresponding photon energies are nearly all in the range 15–60 keV except for a few ‘bad’ spots.

It is known that the structure factor *F_hkl_* is lower for higher orders of *hkl* indices (*e.g.* *F*_113_^2^ / *F*_111_^2^ = 0.53 for Al). Combined with the lower X-ray flux as well as lower detective quantum efficiency at the higher-energy end, the spot intensities for the higher {*hkl*} families are therefore lower. For the relatively small grain #3, these two in combination are more significant, leading to spot intensities indistinguishable from the background for photon energies >60 keV. In contrast, the large volume of grain #2 ensures pronounced spot intensities even for very high {*hkl*} families, which makes them clearly visible above the background intensity. As the X-ray spectrum profile for a given X-ray tube is affected by both the electron accelerating voltage and current, the two parameters can be tuned for different samples to optimize the detectable number and intensity of diffraction spots. For example, to resolve more diffraction spots for small grains with better accuracy, maximizing the fluxes of X-rays with energies in the range 15–60 keV is expected to improve the signal-to-noise ratio for the strongest spots from low *hkl* indices for Al.

## 5. Conclusions   

We have developed a forward simulation model for LabDCT. The model principles are described in detail. A novel approach, by considering diffraction events for each meshed polyhedron in each grain, is used for implementing the model. This polyhedron meshing based approach has the advantage of conforming to grain boundaries, thereby avoiding ‘staircase’ artifacts inherent to voxelized grids. The accuracy of the model has been verified by good agreements between (i) a virtual input grain structure and the reconstructed one based on the simulated diffraction projections of the input structure and (ii) the computed and experimental diffraction spots from a partially recrystallized Al sample.

Based on the results presented for the applications of the model to strain-free materials with grain sizes >40 µm in Laue focusing geometry, it is found that:

(i) Experimental spots, including the weak and overlapped ones, can be retrieved with the assistance of the presented forward simulation model.

(ii) The theoretical maximum completeness, *i.e.* the number of experimentally observed spots divided by the theoretically predicted number of spots, is grain size dependent. For a fixed number of {*hkl*} families, it increases with increasing grain size.

(iii) Diffraction spots from higher-order {*hkl*} families are in general from photons with higher energies and experimentally their visibility is reduced with decreasing grain size.

(iv) For an Al sample characterized with the typical Laue focusing condition, diffraction spots from up to the tenth {*hkl*} family can be seen for a 250 µm grain, while spots from the first four {*hkl*} families are only visible for a 40 µm grain. The diffraction spots from the first four strongest (also most important) {*hkl*} families are mainly from photons with energies in the range 15–60 keV.

Such analysis provides important understanding of LabDCT results and guidelines to optimize experimental parameters, like tuning the X-ray source spectrum profile, according to specific samples. The model can handle any crystal symmetries and any geometries of *L*_sd_ / *L*_ss_. Other input that depends on specific instrumentation such as the X-ray spectrum and detective quantum efficiency of the detector system can be readily tuned and incorporated into the forward simulation model. The versatility and flexibility of the current simulation model make it a useful tool for any LabDCT characterization.

## Figures and Tables

**Figure 9:**
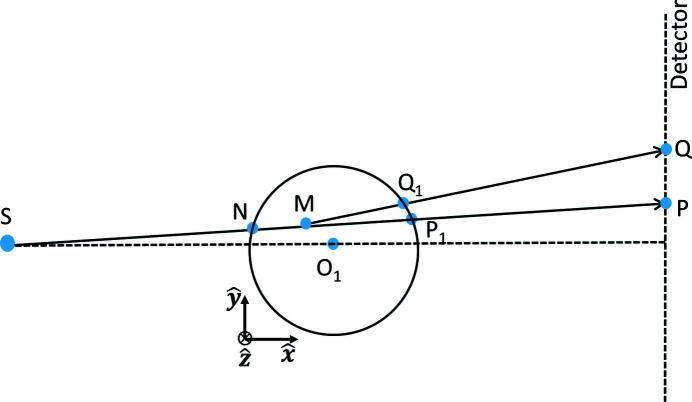
Top view of the sketch for a diffraction event occurring at position *M* inside a cylinder sample. The incoming beam intersects with the cylinder surface at points *N* and *P*_1_. The diffracted beam intersects with the cylinder surface at point *Q*_1_. The center of the intersecting plane is projected at point *O*_1_. Other symbols have the same meanings as in Fig. 1 ▸.

**Figure 10:**
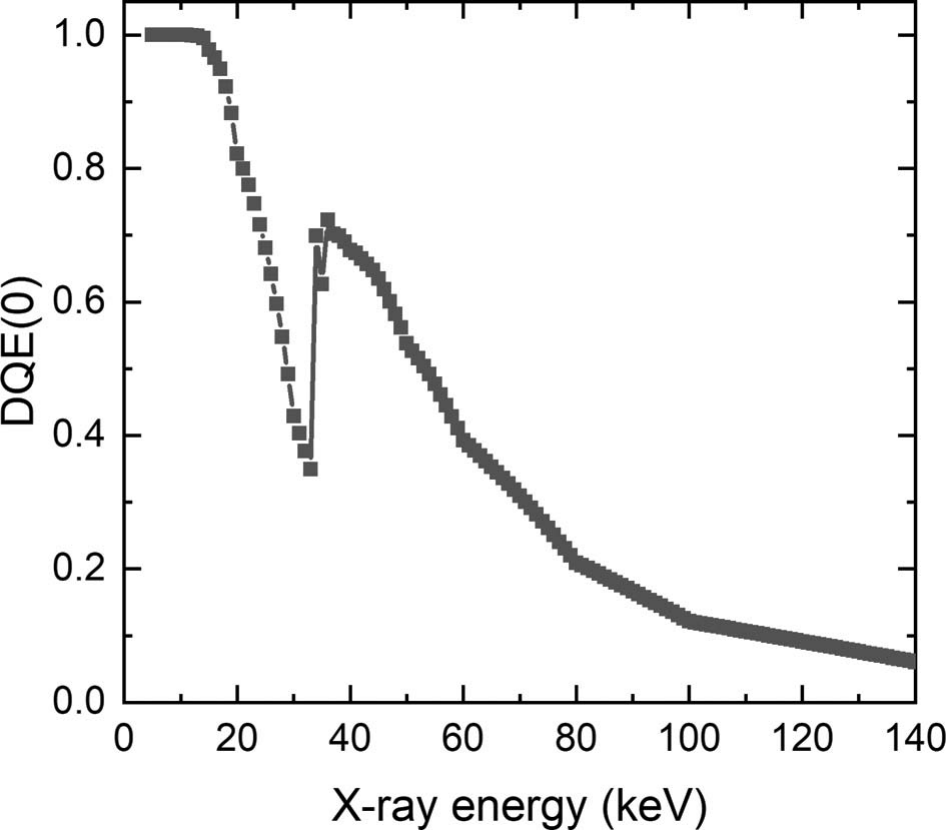
DQE(0) as a function of X-ray energy for a detector system using a 150 µm-thick CsI scintillator.

**Table 1:** Characteristics of the reconstructed grains in the partially recrystallized Al sample Euler angles [φ_1_, Φ, φ_2_] are expressed in Bunge’s convention. Mean values of Euler angles are given for the deformed grain #1. Mean values and standard deviations are listed for the completeness of all voxels within a grain.

Grain ID	[φ_1_, Φ, φ_2_] (°)	Diameter (µm)	Completeness	Observed spot number for the first four {*hkl*} families
#1	[344.29, 23.45, 29.00]	587.8	0.551 ± 0.055	—
#2	[81.02, 9.35, 265.84]	248.5	0.888 ± 0.081	341
#3	[346.06, 15.12, 27.30]	71.2	0.829 ± 0.087	228
#4	[339.90, 24.81, 22.43]	40.6	0.751 ± 0.040	188
#5	[334.07, 27.37, 36.62]	66.3	0.802 ± 0.068	228
#6	[314.57, 14.89, 57.42]	151.0	0.895 ± 0.097	304

## Appendix A Attenuation intensity factor, *A*(*E*)

Sample attenuation is considered to calculate *A*(*E*) in equation (8) for an X-ray energy *E*. As shown in Fig. 9 ▸, the length of the incoming beam path in the sample is  and the length of the diffraction beam path in the sample is . Thus, the total length of the beam attenuated by the sample is . Assuming a cylinder sample shape with a radius of *R*_s_, the cylinder surface can be described by

The intersection point *N* (*x_n_*, *y_n_*, *z_n_*) between the line *SM* and the cylinder surface can be expressed as

where τ_1_ is an unknown parameter, which can be derived by solving equation (9) with (*x*, *y*) substituted by (*x_n_*, *y_n_*). Two solutions may exist for τ_1_, corresponding to the coordinates of *N* and *P*_1_, respectively. The solution of τ_1_ for deriving point *N* is calculated as

To determine , the coordinate of the point *Q*_1_ (intersection of line *MQ* and the sample cylinder surface) has to be derived. The coordinate (*x_q_*_1_, *y_q_*_1_, *z_q_*_1_) of *Q*_1_ can be expressed as

where (*y*_det_, *z*_det_) can be calculated with equation (7) and τ_2_ is an unknown parameter, which can be derived by solving equation (9) with (*x*, *y*) substituted by (*x_q_*_1_, *y_q_*_1_). Similarly, two solutions for τ_2_ may exist. The solution of τ_2_ for deriving point *Q*_1_ can be calculated as

After the coordinates of points *N* and *Q*_1_ are derived,  and  and thus *L*_attenu_ can be readily calculated. Therefore, the attenuation intensity factor *A*(*E*) can be derived:

where μ(*E*)_sample_ is the linear attenuation coefficient of the sample at a photon energy of *E*, which can be retrieved from the NIST X-ray attenuation databases (Hubbell & Seltzer, 2004 ▸).

For the present Al sample that has a roughly square cross section as described in Section 3.2, *A*(*E*) was determined using this method, assuming that the sample is a cylinder with *R*_s_ as the maximum radius of circumcircle of the sample cross section in the *XY* plane. This is expected to lead to only a small error in determining *A*(*E*) for Al.

## Appendix B Detective quantum efficiency, DQE

The performance of a detector can be described by detective quantum efficiency (DQE), which can be expressed by (Jaffray *et al.*, 1995 ▸)

where  and  are the output and input signal-to-noise ratios, respectively. According to Swank (1973 ▸), DQE can be rewritten as

where ∊ is the quantum absorption efficiency of the detector and *I_x_* is the Swank statistical factor characterizing noise increase due to variable X-ray energy absorption.

For a scintillated detector system (which is widely used in modern laboratory X-ray imaging setups), the quantum absorption efficiency at a particular X-ray energy, ∊(*E*), can be calculated as the absorption by the scintillator:

where μ(*E*)_scintillator_ is the linear attenuation coefficient at an X-ray energy of *E* and *L*_scintillator_ is the scintillator’s thickness.

The Swank factor *I_x_* is calculated from the absorbed X-ray energy distribution (AED), which describes the probability per unit energy that an incident X-ray will deposit a certain energy within the detector. Since AED cannot be measured directly, it is best estimated by Monte Carlo simulations (Jaffray *et al.*, 1995 ▸). AED is shown to be dependent on spatial frequency related to random variations in absorbed energy, incident X-ray energy and scintillator materials.

For the present study, we use zero-frequency data of *I_x_* calculated from Monte Carlo simulations (Hajdok *et al.*, 2008 ▸). Since *I_x_* decreases with increasing spatial frequency, *I_x_* derived from zero frequency represents its upper limit. Combining the *I_x_* data and equations (16) and (17), DQE at zero frequency as a function of X-ray energy was calculated for a CsI scintillator with a thickness of 150 µm, which is the same as used in our LabDCT experiment (shown in Fig. 10 ▸). The figure shows that DQE(0) decreases with increasing X-ray energy until two partial recoveries occurring at 33.17 keV (*K* edge of iodine) and 35.98 keV (*K* edge of caesium), after which a consecutive decrease goes on. An increase in the scintillator’s thickness enhances DQE(0) due to an increased photon absorption ability. It should be pointed out that there are other factors influencing DQE, such as absorption by other materials like the scintillator’s substrate, non-energy-absorbing scattering and non-zero spatial frequency (Drangova & Rowlands, 1986 ▸). However, those factors are expected to play less significant roles and thus are not considered here.
